# Supplementary material for: A metagenomic insight into freshwater methane-utilizing communities and evidence for cooperation between the Methylococcaceae and the Methylophilaceae
Source: PeerJ. 2013 Feb 19;1:e23. doi: 10.7717/peerj.23 (PMC3628875; doi:10.7717/peerj.23)
Supplement: Supplemental Figure 1 [file peerj-01-23-s001.pdf]

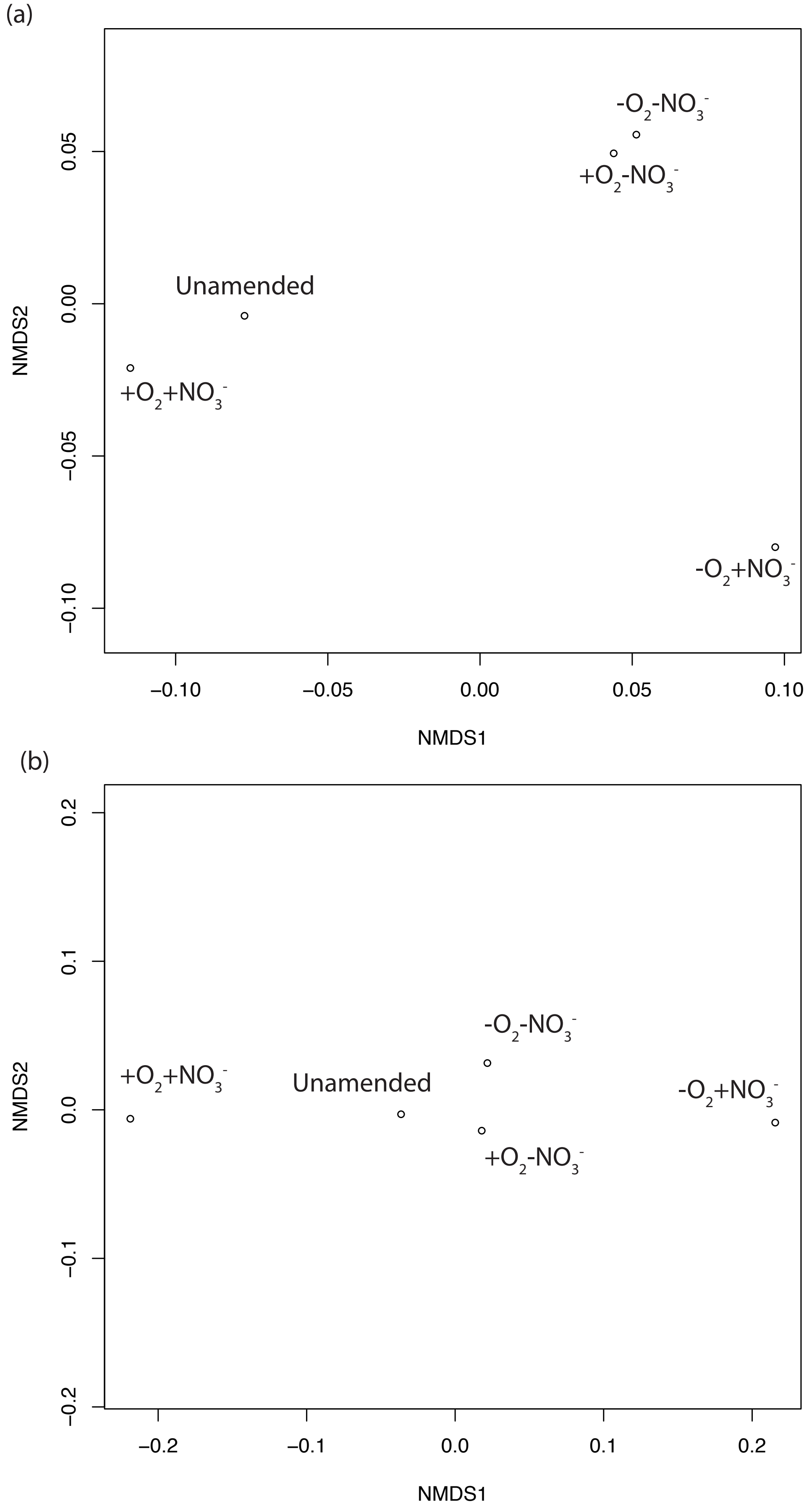

Supplemental Figure 1. Non-metric multidimensional scaling (NMDS) of Bray-Curtis dissimilarity matrix constructed from (a) taxonomical assignments at the family level by BLAST best hit and (b) COG functional assignments for predicted protein products. Taxonomical and COG assignments were performed with IMG/M. NMDS was computed with vegan:

Oksanen J, Blanchet FG, Kindt R, Legendre P, O'Hara RB, Simpson GL, Solymos P, Stevens MHH and Wagner H. 2011. vegan: Community Ecology Package. R package version 1.17-6. http://CRAN.R-project.org/package=vegan
