# Supplementary material for: A metagenomic insight into freshwater methane-utilizing communities and evidence for cooperation between the Methylococcaceae and the Methylophilaceae
Source: PeerJ. 2013 Feb 19;1:e23. doi: 10.7717/peerj.23 (PMC3628875; doi:10.7717/peerj.23)
Supplement: Supplemental Tables 2 and 3 [file peerj-01-23-s002.docx]

Supplemental Table 2. Relative abundance of *mmoX** genes. Actual numbers of genes are shown.

| Taxon | Unamended | +O_2_-NO_3_^-^ | +O_2_+NO_3_^-^ | -O_2_-NO_3_^-^ | -O_2_+NO_3_^-^ |
| --- | --- | --- | --- | --- | --- |
| *Methylococcaceae* | 0 | 5 | 3 | 0 | 0 |
| *Methylocystaceae* | 1 | 13 | 0 | 0 | 0 |

**mmoX* encodes the alpha subunit of soluble methane monooxygenase.

Supplemental Table 3. Relative abundance of *mxaF/xoxF** genes representing *Methylococcaceae, Methylophilaceae* and *Methylocystaceae.* Actual numbers of genes are shown.

| Enzyme/microcosm | Unamended | +O_2_-NO_3_^-^ | +O_2_+NO_3_^-^ | -O_2_-NO_3_^-^ | O_2_+NO_3_^-^ |
| --- | --- | --- | --- | --- | --- |
| *Methylococcaceae* MxaF | 2 | 12 | 9 | 0 | 3 |
| *Methylococcaceae* XoxF | 27 | 41 | 17 | 4 | 6 |
| *Methylophilaceae* MxaF | 0 | 2 | 0 | 0 | 0 |
| *Methylophilaceae* XoxF | 19 | 92 | 37 | 0 | 4 |
| *Methylocystaceae* MxaF | 0 | 16 | 0 | 0 | 0 |
| *Methylocystaceae* XoxF | 0 | 15 | 0 | 0 | 0 |

**mxaF* encodes the large subunit of methanol dehydrogenase. *xoxF* is a homolog of *mxaF.*
