# Supplementary material for: A metagenomic insight into freshwater methane-utilizing communities and evidence for cooperation between the Methylococcaceae and the Methylophilaceae
Source: PeerJ. 2013 Feb 19;1:e23. doi: 10.7717/peerj.23 (PMC3628875; doi:10.7717/peerj.23)
Supplement: Supplemental Tables 4--8 [file peerj-01-23-s003.docx]

Supplemental Table 4. Count of genes annotated as NO_3_^-^ reductase classified at the genus level.

| Genus | Un-amended | +O_2_-NO_3_^-^ | +O_2_+NO_3_^-^ | -O_2_-NO_3_^-^ | -O_2_+NO_3_^-^ |
| --- | --- | --- | --- | --- | --- |
| Methylobacter | 178 | 210 | 86 | 1 | 27 |
| Methylotenera | 19 | 90 | 41 | 2 | 13 |
| uncultured bacterium | 44 | 24 | 6 | 0 | 37 |
| Methylophaga | 19 | 38 | 16 | 0 | 7 |
| Sterolibacterium | 40 | 8 | 1 | 1 | 22 |
| Streptomyces | 43 | 5 | 1 | 0 | 15 |
| Rhodoferax | 19 | 16 | 9 | 0 | 19 |
| Thiobacillus | 9 | 39 | 8 | 0 | 1 |
| uncultured prokaryote | 26 | 3 | 0 | 3 | 12 |
| Anaeromyxobacter | 23 | 7 | 0 | 1 | 12 |
| Beggiatoa | 18 | 6 | 2 | 2 | 12 |
| Burkholderia | 21 | 1 | 6 | 2 | 10 |
| Methyloversatilis | 14 | 6 | 2 | 1 | 15 |
| Polaromonas | 9 | 10 | 5 | 1 | 13 |
| Geobacter | 21 | 3 | 1 | 1 | 11 |
| Desulfococcus | 17 | 4 | 0 | 5 | 7 |
| Acidovorax | 10 | 7 | 7 | 0 | 8 |
| Aromatoleum | 21 | 5 | 2 | 0 | 2 |
| Candidatus Nitrospira defluvii | 18 | 1 | 0 | 2 | 6 |
| uncultured candidate division OP1 bacterium | 10 | 2 | 1 | 1 | 10 |
| uncultured microorganism | 10 | 3 | 1 | 1 | 8 |
| Nitrobacter | 6 | 6 | 0 | 0 | 10 |
| Hydrogenophaga | 11 | 2 | 1 | 1 | 6 |
| Chthoniobacter | 6 | 6 | 0 | 1 | 6 |
| Dechlorosoma | 9 | 1 | 2 | 0 | 5 |
| endosymbiont | 5 | 11 | 1 | 0 | 0 |
| Shewanella | 8 | 5 | 4 | 0 | 0 |
| Thioalkalivibrio | 5 | 0 | 6 | 0 | 6 |
| gamma | 9 | 0 | 1 | 0 | 6 |
| Hyphomicrobium | 5 | 5 | 1 | 0 | 5 |
| Methylibium | 6 | 1 | 2 | 0 | 7 |
| Methylovorus | 1 | 15 | 0 | 0 | 0 |
| Pseudomonas | 4 | 2 | 0 | 1 | 9 |
| Methylobacterium | 6 | 3 | 1 | 1 | 4 |
| Methylocystis | 0 | 13 | 2 | 0 | 0 |
| NC10 | 8 | 1 | 0 | 0 | 6 |
| Desulfurispirillum | 6 | 0 | 1 | 0 | 7 |
| Photobacterium | 9 | 1 | 0 | 0 | 4 |
| Desulfobacterium | 10 | 0 | 0 | 1 | 2 |
| Sorangium | 5 | 4 | 1 | 0 | 3 |
| Azoarcus | 3 | 0 | 2 | 0 | 7 |
| Chryseobacterium | 8 | 4 | 0 | 0 | 0 |
| Herminiimonas | 3 | 0 | 0 | 0 | 9 |
| Mycobacterium | 4 | 3 | 0 | 0 | 5 |
| Plesiocystis | 6 | 2 | 0 | 0 | 4 |
| Rhodothermus | 12 | 0 | 0 | 0 | 0 |
| Bacillus | 1 | 9 | 0 | 0 | 1 |
| Bradyrhizobium | 3 | 7 | 0 | 0 | 1 |
| Candidatus Accumulibacter phosphatis clade IIA str. UW-1 | 5 | 1 | 0 | 1 | 4 |
| Candidatus Kuenenia stuttgartiensis | 6 | 0 | 0 | 5 | 0 |
| Delftia | 5 | 2 | 1 | 1 | 2 |
| Thauera | 3 | 2 | 1 | 0 | 5 |
| Burkholderiales | 3 | 1 | 3 | 0 | 3 |
| Marinithermus | 6 | 2 | 0 | 0 | 2 |
| Moritella | 2 | 5 | 2 | 0 | 1 |
| Thermus | 6 | 1 | 0 | 0 | 3 |
| Thiocapsa | 5 | 2 | 0 | 0 | 3 |
| Variovorax | 2 | 2 | 3 | 0 | 3 |
| Mesorhizobium | 2 | 5 | 0 | 0 | 2 |
| Nitrococcus | 3 | 1 | 2 | 2 | 1 |
| Ralstonia | 3 | 1 | 2 | 0 | 3 |
| Rubrobacter | 6 | 0 | 0 | 0 | 3 |
| Afipia | 3 | 2 | 1 | 0 | 2 |
| Bordetella | 4 | 3 | 0 | 0 | 1 |
| Caldilinea | 4 | 1 | 0 | 0 | 3 |
| Dechloromonas | 5 | 1 | 0 | 0 | 2 |
| Brevundimonas | 4 | 2 | 1 | 0 | 0 |
| Candidatus Solibacter usitatus Ellin6076 | 6 | 0 | 0 | 0 | 1 |
| Paracoccus | 0 | 2 | 2 | 0 | 3 |
| planctomycete | 6 | 0 | 0 | 0 | 1 |
| Saccharophagus | 4 | 2 | 1 | 0 | 0 |
| Arthrobacter | 1 | 2 | 1 | 0 | 2 |
| Comamonas | 1 | 5 | 0 | 0 | 0 |
| Conexibacter | 2 | 1 | 0 | 0 | 3 |
| Desulfosporosinus | 2 | 0 | 0 | 0 | 4 |
| Meiothermus | 2 | 1 | 1 | 0 | 2 |
| Methylocella | 1 | 2 | 1 | 0 | 2 |
| Arthrospira | 2 | 1 | 0 | 1 | 1 |
| Citreicella | 0 | 1 | 0 | 0 | 4 |
| Herpetosiphon | 2 | 0 | 0 | 0 | 3 |
| Oxalobacteraceae | 0 | 0 | 0 | 0 | 5 |
| uncultured organism | 1 | 1 | 1 | 0 | 2 |
| Azotobacter | 4 | 0 | 0 | 0 | 0 |
| Brachymonas | 4 | 0 | 0 | 0 | 0 |
| Bradyrhizobiaceae | 2 | 1 | 1 | 0 | 0 |
| Brucella | 2 | 1 | 0 | 0 | 1 |
| Campylobacter | 2 | 0 | 0 | 0 | 2 |
| Candidatus Caldiarchaeum subterraneum | 2 | 1 | 0 | 0 | 1 |
| Cellulomonas | 0 | 0 | 3 | 0 | 1 |
| Escherichia | 4 | 0 | 0 | 0 | 0 |
| Haliangium | 3 | 0 | 0 | 0 | 1 |
| Hydrogenobacter | 2 | 1 | 0 | 0 | 1 |
| Lyngbya | 4 | 0 | 0 | 0 | 0 |
| Maribacter | 2 | 0 | 0 | 0 | 2 |
| Niabella | 0 | 2 | 0 | 0 | 2 |
| Niastella | 0 | 4 | 0 | 0 | 0 |
| Oceanithermus | 1 | 1 | 0 | 0 | 2 |
| Oligotropha | 1 | 0 | 0 | 0 | 3 |
| Paenibacillus | 0 | 0 | 0 | 4 | 0 |
| Persephonella | 2 | 0 | 1 | 0 | 1 |
| Rhodopseudomonas | 0 | 3 | 1 | 0 | 0 |
| Sphingobacterium | 0 | 0 | 0 | 0 | 4 |
| Stenotrophomonas | 1 | 0 | 0 | 0 | 3 |
| Thiomicrospira | 2 | 1 | 1 | 0 | 0 |
| Amycolicicoccus | 0 | 0 | 0 | 0 | 3 |
| Azorhizobium | 1 | 1 | 0 | 1 | 0 |
| Candidatus Chloracidobacterium thermophilum B | 2 | 0 | 0 | 0 | 1 |
| Cupriavidus | 2 | 1 | 0 | 0 | 0 |
| Desulfovibrio | 0 | 0 | 0 | 1 | 2 |
| Dictyostelium | 3 | 0 | 0 | 0 | 0 |
| Halomonas | 1 | 0 | 0 | 0 | 2 |
| Janthinobacterium | 0 | 1 | 0 | 0 | 2 |
| Leptothrix | 0 | 1 | 0 | 0 | 2 |
| Lutiella | 1 | 0 | 0 | 1 | 1 |
| Pedosphaera | 2 | 1 | 0 | 0 | 0 |
| Pseudogulbenkiania | 1 | 0 | 0 | 0 | 2 |
| Rheinheimera | 1 | 0 | 0 | 1 | 1 |
| Saccharomonospora | 2 | 1 | 0 | 0 | 0 |
| Salmonella | 3 | 0 | 0 | 0 | 0 |
| Sulfurihydrogenibium | 3 | 0 | 0 | 0 | 0 |
| Achromobacter | 1 | 0 | 1 | 0 | 0 |
| Archaeoglobus | 2 | 0 | 0 | 0 | 0 |
| Azospirillum | 1 | 1 | 0 | 0 | 0 |
| Caulobacter | 1 | 1 | 0 | 0 | 0 |
| Denitrovibrio | 0 | 0 | 0 | 0 | 2 |
| Desulfitobacterium | 0 | 0 | 0 | 0 | 2 |
| Emticicia | 1 | 1 | 0 | 0 | 0 |
| Ferroglobus | 1 | 0 | 0 | 0 | 1 |
| Flavobacterium | 0 | 1 | 0 | 0 | 1 |
| Haloferax | 2 | 0 | 0 | 0 | 0 |
| Halorubrum | 1 | 0 | 0 | 0 | 1 |
| Helicobacter | 1 | 1 | 0 | 0 | 0 |
| Herbaspirillum | 1 | 0 | 1 | 0 | 0 |
| Hydrogenivirga | 1 | 0 | 0 | 0 | 1 |
| Hylemonella | 0 | 0 | 2 | 0 | 0 |
| Kangiella | 1 | 1 | 0 | 0 | 0 |
| Methylomonas | 0 | 1 | 1 | 0 | 0 |
| Myxococcus | 1 | 0 | 0 | 0 | 1 |
| Nocardioides | 0 | 0 | 0 | 0 | 2 |
| Opitutus | 0 | 1 | 0 | 0 | 1 |
| Phenylobacterium | 0 | 2 | 0 | 0 | 0 |
| Planctomyces | 1 | 0 | 0 | 0 | 1 |
| Providencia | 2 | 0 | 0 | 0 | 0 |
| Pseudonocardia | 1 | 1 | 0 | 0 | 0 |
| Psychrobacter | 1 | 0 | 0 | 0 | 1 |
| Rhodanobacter | 0 | 0 | 0 | 0 | 2 |
| Ruegeria | 1 | 1 | 0 | 0 | 0 |
| Saccharopolyspora | 1 | 0 | 0 | 0 | 1 |
| Sideroxydans | 0 | 2 | 0 | 0 | 0 |
| Sphaerobacter | 0 | 0 | 0 | 1 | 1 |
| Stigmatella | 1 | 0 | 0 | 0 | 1 |
| Streptosporangium | 2 | 0 | 0 | 0 | 0 |
| Sulfuricurvum | 1 | 0 | 0 | 0 | 1 |
| Sulfurovum | 0 | 1 | 0 | 0 | 1 |
| Acaryochloris | 0 | 0 | 0 | 0 | 1 |
| Acetonema | 1 | 0 | 0 | 0 | 0 |
| Acidithiobacillus | 0 | 0 | 1 | 0 | 0 |
| Acidothermus | 1 | 0 | 0 | 0 | 0 |
| Actinosynnema | 1 | 0 | 0 | 0 | 0 |
| Aeromonas | 0 | 0 | 0 | 0 | 1 |
| Agrobacterium | 1 | 0 | 0 | 0 | 0 |
| alpha | 1 | 0 | 0 | 0 | 0 |
| Arcobacter | 1 | 0 | 0 | 0 | 0 |
| Beutenbergia | 1 | 0 | 0 | 0 | 0 |
| Candidatus Vesicomyosocius okutanii HA | 1 | 0 | 0 | 0 | 0 |
| Chelativorans | 0 | 1 | 0 | 0 | 0 |
| Chromobacterium | 0 | 0 | 0 | 0 | 1 |
| Chromohalobacter | 1 | 0 | 0 | 0 | 0 |
| Clostridiales | 0 | 0 | 0 | 0 | 1 |
| Congregibacter | 0 | 1 | 0 | 0 | 0 |
| Curvibacter | 0 | 1 | 0 | 0 | 0 |
| Cyanothece | 0 | 1 | 0 | 0 | 0 |
| Deferribacter | 0 | 0 | 1 | 0 | 0 |
| Deinococcus | 1 | 0 | 0 | 0 | 0 |
| Denitromonas | 0 | 1 | 0 | 0 | 0 |
| Desulfobacca | 1 | 0 | 0 | 0 | 0 |
| Desulfohalobium | 1 | 0 | 0 | 0 | 0 |
| Dickeya | 0 | 1 | 0 | 0 | 0 |
| Eubacterium | 0 | 0 | 0 | 0 | 1 |
| Gallibacterium | 1 | 0 | 0 | 0 | 0 |
| Geobacillus | 0 | 0 | 0 | 0 | 1 |
| Geodermatophilus | 0 | 0 | 0 | 0 | 1 |
| Gluconacetobacter | 1 | 0 | 0 | 0 | 0 |
| Gordonia | 0 | 0 | 0 | 0 | 1 |
| Hahella | 1 | 0 | 0 | 0 | 0 |
| Haliscomenobacter | 0 | 0 | 0 | 0 | 1 |
| Haloarcula | 1 | 0 | 0 | 0 | 0 |
| Halogeometricum | 1 | 0 | 0 | 0 | 0 |
| Haloterrigena | 1 | 0 | 0 | 0 | 0 |
| Holophaga | 1 | 0 | 0 | 0 | 0 |
| Hydrogenobaculum | 0 | 0 | 0 | 0 | 1 |
| Hyphomonas | 0 | 0 | 0 | 0 | 1 |
| Intrasporangium | 1 | 0 | 0 | 0 | 0 |
| Janibacter | 0 | 0 | 0 | 0 | 1 |
| Joostella | 0 | 1 | 0 | 0 | 0 |
| Kingella | 1 | 0 | 0 | 0 | 0 |
| Kribbella | 0 | 0 | 0 | 0 | 1 |
| Ktedonobacter | 0 | 1 | 0 | 0 | 0 |
| Laribacter | 1 | 0 | 0 | 0 | 0 |
| Lentisphaera | 0 | 1 | 0 | 0 | 0 |
| Leptonema | 1 | 0 | 0 | 0 | 0 |
| Limnobacter | 0 | 1 | 0 | 0 | 0 |
| Magnetospirillum | 0 | 0 | 0 | 0 | 1 |
| Maricaulis | 1 | 0 | 0 | 0 | 0 |
| Metallosphaera | 1 | 0 | 0 | 0 | 0 |
| Micromonospora | 0 | 0 | 0 | 0 | 1 |
| Mucilaginibacter | 0 | 1 | 0 | 0 | 0 |
| Natranaerobius | 0 | 0 | 0 | 0 | 1 |
| Nitratifractor | 1 | 0 | 0 | 0 | 0 |
| Nocardia | 1 | 0 | 0 | 0 | 0 |
| Novosphingobium | 1 | 0 | 0 | 0 | 0 |
| Ochrobactrum | 0 | 1 | 0 | 0 | 0 |
| Octadecabacter | 0 | 0 | 1 | 0 | 0 |
| Opitutaceae | 1 | 0 | 0 | 0 | 0 |
| Oscillochloris | 0 | 0 | 0 | 0 | 1 |
| Pectobacterium | 0 | 1 | 0 | 0 | 0 |
| Pseudoxanthomonas | 0 | 0 | 0 | 1 | 0 |
| Pyrobaculum | 1 | 0 | 0 | 0 | 0 |
| Ramlibacter | 0 | 0 | 1 | 0 | 0 |
| Roseovarius | 0 | 0 | 0 | 0 | 1 |
| Selenomonas | 0 | 1 | 0 | 0 | 0 |
| Serratia | 1 | 0 | 0 | 0 | 0 |
| Solitalea | 0 | 1 | 0 | 0 | 0 |
| Sphingobium | 0 | 0 | 0 | 0 | 1 |
| Sphingomonas | 0 | 0 | 0 | 0 | 1 |
| Spirosoma | 0 | 1 | 0 | 0 | 0 |
| Sulfurimonas | 0 | 0 | 0 | 0 | 1 |
| Sulfurospirillum | 0 | 0 | 0 | 1 | 0 |
| Sutterella | 0 | 1 | 0 | 0 | 0 |
| Synechococcus | 0 | 0 | 0 | 0 | 1 |
| Syntrophobacter | 0 | 0 | 0 | 0 | 1 |
| Terriglobus | 0 | 1 | 0 | 0 | 0 |
| Thalassiosira | 1 | 0 | 0 | 0 | 0 |
| Thiorhodococcus | 1 | 0 | 0 | 0 | 0 |
| Thiothrix | 1 | 0 | 0 | 0 | 0 |
| Verminephrobacter | 0 | 0 | 0 | 1 | 0 |
| Verrucomicrobium | 0 | 1 | 0 | 0 | 0 |
| Xanthobacter | 0 | 0 | 0 | 0 | 1 |
| Xanthomonas | 0 | 1 | 0 | 0 | 0 |

Supplemental Table 5. Count of genes annotated as NO_2_^-^ reductase classified at the genus level.

| Genus | Un-amended | +O_2_-NO_3_^-^ | +O_2_+NO_3_^-^ | -O_2_-NO_3_^-^ | -O_2_+NO_3_^-^ |
| --- | --- | --- | --- | --- | --- |
| Methylobacter | 88 | 122 | 52 | 3 | 17 |
| Methylotenera | 17 | 87 | 37 | 0 | 6 |
| uncultured bacterium | 23 | 15 | 1 | 1 | 16 |
| Candidatus Nitrospira defluvii | 41 | 4 | 1 | 0 | 8 |
| Methylomonas | 9 | 11 | 9 | 0 | 2 |
| Methylomicrobium | 4 | 16 | 7 | 0 | 0 |
| uncultured bacterium 2303 | 10 | 5 | 1 | 1 | 9 |
| Methylocystis | 2 | 20 | 1 | 0 | 0 |
| Chthoniobacter | 7 | 7 | 1 | 0 | 5 |
| Methylovorus | 2 | 12 | 3 | 0 | 2 |
| Oceanimonas | 3 | 6 | 6 | 0 | 0 |
| Pelobacter | 9 | 1 | 2 | 0 | 3 |
| Ralstonia | 7 | 1 | 1 | 0 | 6 |
| Sideroxydans | 8 | 4 | 0 | 0 | 3 |
| Acidovorax | 4 | 3 | 3 | 0 | 4 |
| Conexibacter | 6 | 1 | 0 | 0 | 7 |
| uncultured microorganism | 5 | 2 | 0 | 1 | 6 |
| Burkholderiales | 5 | 4 | 1 | 0 | 2 |
| Leptothrix | 5 | 3 | 1 | 0 | 3 |
| Turneriella | 1 | 6 | 2 | 0 | 2 |
| Bradyrhizobium | 4 | 4 | 0 | 0 | 2 |
| Pusillimonas | 5 | 4 | 1 | 0 | 0 |
| Burkholderia | 2 | 4 | 2 | 0 | 1 |
| Candidatus Accumulibacter phosphatis clade IIA str. UW-1 | 4 | 2 | 0 | 0 | 3 |
| Haliangium | 3 | 0 | 0 | 0 | 6 |
| Neisseria | 2 | 4 | 3 | 0 | 0 |
| Opitutus | 4 | 1 | 1 | 1 | 2 |
| uncultured bacterium 888 | 2 | 1 | 0 | 0 | 6 |
| Anaeromyxobacter | 5 | 0 | 0 | 1 | 2 |
| Maribacter | 6 | 0 | 1 | 0 | 1 |
| Polaromonas | 2 | 1 | 5 | 0 | 0 |
| Thiobacillus | 5 | 2 | 0 | 0 | 1 |
| Afipia | 1 | 4 | 2 | 0 | 0 |
| Dechloromonas | 4 | 1 | 0 | 0 | 2 |
| Dechlorosoma | 3 | 2 | 0 | 0 | 2 |
| Desulfovibrio | 6 | 0 | 0 | 0 | 1 |
| Herpetosiphon | 2 | 1 | 0 | 0 | 4 |
| Ignavibacterium | 1 | 1 | 0 | 4 | 1 |
| Lutiella | 3 | 4 | 0 | 0 | 0 |
| Streptomyces | 5 | 1 | 0 | 0 | 1 |
| Anaerolinea | 4 | 0 | 0 | 0 | 2 |
| Candidatus Nitrosoarchaeum koreensis MY1 | 1 | 4 | 0 | 0 | 1 |
| Chloroflexus | 1 | 2 | 2 | 0 | 1 |
| Methyloversatilis | 2 | 2 | 0 | 0 | 2 |
| planctomycete | 4 | 0 | 1 | 1 | 0 |
| Psychrobacter | 0 | 5 | 1 | 0 | 0 |
| Sorangium | 3 | 0 | 0 | 0 | 3 |
| Thermaerobacter | 2 | 2 | 0 | 0 | 2 |
| Kangiella | 3 | 1 | 0 | 0 | 1 |
| Naegleria | 4 | 0 | 0 | 0 | 1 |
| Nitrosomonas | 4 | 0 | 0 | 1 | 0 |
| Synechococcus | 3 | 0 | 0 | 0 | 2 |
| uncultured organism | 2 | 0 | 1 | 0 | 2 |
| Aromatoleum | 2 | 2 | 0 | 0 | 0 |
| Candidatus Solibacter usitatus Ellin6076 | 2 | 0 | 0 | 1 | 1 |
| Coraliomargarita | 1 | 0 | 0 | 0 | 3 |
| Hahella | 1 | 1 | 0 | 1 | 1 |
| Hyphomicrobium | 0 | 1 | 0 | 0 | 3 |
| Marinithermus | 0 | 2 | 0 | 0 | 2 |
| Methylophaga | 0 | 1 | 1 | 1 | 1 |
| Planctomyces | 2 | 2 | 0 | 0 | 0 |
| Pseudoalteromonas | 1 | 2 | 1 | 0 | 0 |
| Rubrivivax | 1 | 2 | 0 | 0 | 1 |
| Singulisphaera | 0 | 0 | 0 | 0 | 4 |
| Sulfurimonas | 1 | 0 | 0 | 0 | 3 |
| Azoarcus | 2 | 0 | 0 | 0 | 1 |
| Collimonas | 0 | 2 | 0 | 0 | 1 |
| Curvibacter | 1 | 0 | 1 | 0 | 1 |
| delta | 3 | 0 | 0 | 0 | 0 |
| endosymbiont | 3 | 0 | 0 | 0 | 0 |
| Flavobacterium | 1 | 0 | 0 | 1 | 1 |
| Methylibium | 2 | 0 | 0 | 0 | 1 |
| Nitrosococcus | 2 | 1 | 0 | 0 | 0 |
| Nitrosopumilus | 0 | 0 | 0 | 0 | 3 |
| Novosphingobium | 0 | 2 | 1 | 0 | 0 |
| Oscillochloris | 1 | 0 | 0 | 0 | 2 |
| Pedosphaera | 2 | 0 | 0 | 0 | 1 |
| Salinisphaera | 1 | 0 | 0 | 0 | 2 |
| Symbiobacterium | 0 | 2 | 1 | 0 | 0 |
| Syntrophobacter | 2 | 1 | 0 | 0 | 0 |
| Thauera | 1 | 0 | 0 | 0 | 2 |
| Variovorax | 0 | 0 | 2 | 0 | 1 |
| Verrucomicrobium | 0 | 2 | 1 | 0 | 0 |
| Alicycliphilus | 2 | 0 | 0 | 0 | 0 |
| Alteromonas | 2 | 0 | 0 | 0 | 0 |
| Bdellovibrio | 1 | 1 | 0 | 0 | 0 |
| Blastopirellula | 2 | 0 | 0 | 0 | 0 |
| Bradyrhizobiaceae | 0 | 2 | 0 | 0 | 0 |
| Caldithrix | 1 | 1 | 0 | 0 | 0 |
| Candidatus Nitrosoarchaeum limnia SFB1 | 0 | 0 | 0 | 1 | 1 |
| Capnocytophaga | 0 | 2 | 0 | 0 | 0 |
| Caulobacter | 1 | 1 | 0 | 0 | 0 |
| Desulfarculus | 2 | 0 | 0 | 0 | 0 |
| Desulfobacula | 1 | 0 | 0 | 1 | 0 |
| Desulfosporosinus | 1 | 0 | 0 | 0 | 1 |
| Geobacter | 1 | 0 | 0 | 0 | 1 |
| Holophaga | 0 | 1 | 0 | 0 | 1 |
| Ilyobacter | 2 | 0 | 0 | 0 | 0 |
| Marinobacter | 1 | 0 | 0 | 0 | 1 |
| Methanocella | 1 | 0 | 0 | 0 | 1 |
| Methylocella | 2 | 0 | 0 | 0 | 0 |
| Methylococcus | 0 | 0 | 0 | 0 | 2 |
| Methylosinus | 0 | 2 | 0 | 0 | 0 |
| Microvirga | 1 | 0 | 1 | 0 | 0 |
| Moraxella | 0 | 1 | 1 | 0 | 0 |
| NC10 | 1 | 0 | 0 | 0 | 1 |
| Niastella | 1 | 1 | 0 | 0 | 0 |
| Nitrosospira | 2 | 0 | 0 | 0 | 0 |
| Oligotropha | 1 | 0 | 1 | 0 | 0 |
| Paenibacillus | 1 | 0 | 0 | 1 | 0 |
| Pirellula | 1 | 0 | 0 | 0 | 1 |
| Polymorphum | 0 | 0 | 1 | 0 | 1 |
| Prevotella | 1 | 0 | 0 | 0 | 1 |
| Pseudomonas | 0 | 0 | 1 | 0 | 1 |
| Rhodoferax | 2 | 0 | 0 | 0 | 0 |
| Rhodopseudomonas | 1 | 1 | 0 | 0 | 0 |
| Rhodothermus | 1 | 1 | 0 | 0 | 0 |
| SAR324 | 0 | 2 | 0 | 0 | 0 |
| Shewanella | 1 | 0 | 0 | 1 | 0 |
| Simonsiella | 2 | 0 | 0 | 0 | 0 |
| Solitalea | 0 | 0 | 0 | 1 | 1 |
| Thermus | 1 | 0 | 0 | 0 | 1 |
| Thiomonas | 1 | 1 | 0 | 0 | 0 |
| Achromobacter | 1 | 0 | 0 | 0 | 0 |
| Alkalilimnicola | 0 | 1 | 0 | 0 | 0 |
| Amycolatopsis | 1 | 0 | 0 | 0 | 0 |
| Azorhizobium | 0 | 1 | 0 | 0 | 0 |
| Azospirillum | 0 | 1 | 0 | 0 | 0 |
| Brachymonas | 0 | 1 | 0 | 0 | 0 |
| Caldilinea | 1 | 0 | 0 | 0 | 0 |
| Candidatus Koribacter versatilis Ellin345 | 0 | 0 | 0 | 1 | 0 |
| Cardiobacterium | 0 | 1 | 0 | 0 | 0 |
| Catenulispora | 1 | 0 | 0 | 0 | 0 |
| Chelativorans | 1 | 0 | 0 | 0 | 0 |
| Clostridium | 1 | 0 | 0 | 0 | 0 |
| Comamonas | 0 | 1 | 0 | 0 | 0 |
| Crassostrea | 1 | 0 | 0 | 0 | 0 |
| Cryptobacterium | 1 | 0 | 0 | 0 | 0 |
| Cupriavidus | 0 | 0 | 0 | 0 | 1 |
| Cylindrospermopsis | 0 | 1 | 0 | 0 | 0 |
| Desulfitobacterium | 0 | 0 | 0 | 0 | 1 |
| Desulfotomaculum | 0 | 0 | 0 | 0 | 1 |
| Dyadobacter | 0 | 0 | 0 | 0 | 1 |
| Eggerthella | 1 | 0 | 0 | 0 | 0 |
| Ferroglobus | 1 | 0 | 0 | 0 | 0 |
| Flexistipes | 1 | 0 | 0 | 0 | 0 |
| Frankia | 0 | 0 | 0 | 0 | 1 |
| Haliscomenobacter | 0 | 0 | 0 | 0 | 1 |
| Haloferax | 1 | 0 | 0 | 0 | 0 |
| Halogeometricum | 0 | 1 | 0 | 0 | 0 |
| Halomicrobium | 0 | 0 | 0 | 0 | 1 |
| Haloterrigena | 0 | 1 | 0 | 0 | 0 |
| Herminiimonas | 0 | 0 | 0 | 0 | 1 |
| Hydrogenobacter | 0 | 0 | 0 | 0 | 1 |
| Jonesia | 0 | 1 | 0 | 0 | 0 |
| Kingella | 0 | 1 | 0 | 0 | 0 |
| Klebsiella | 0 | 1 | 0 | 0 | 0 |
| Ktedonobacter | 0 | 0 | 0 | 1 | 0 |
| Labrenzia | 0 | 0 | 0 | 0 | 1 |
| Lautropia | 0 | 0 | 0 | 0 | 1 |
| Legionella | 0 | 0 | 0 | 1 | 0 |
| Marinomonas | 0 | 1 | 0 | 0 | 0 |
| Meiothermus | 1 | 0 | 0 | 0 | 0 |
| Metallosphaera | 0 | 0 | 0 | 0 | 1 |
| Methanobrevibacter | 0 | 0 | 0 | 1 | 0 |
| Methanohalobium | 0 | 0 | 0 | 1 | 0 |
| Methylobacillus | 1 | 0 | 0 | 0 | 0 |
| Mucilaginibacter | 0 | 1 | 0 | 0 | 0 |
| Mycobacterium | 1 | 0 | 0 | 0 | 0 |
| Myroides | 0 | 0 | 1 | 0 | 0 |
| Nakamurella | 0 | 0 | 0 | 0 | 1 |
| Nitrobacter | 0 | 1 | 0 | 0 | 0 |
| Nocardia | 1 | 0 | 0 | 0 | 0 |
| Nodularia | 1 | 0 | 0 | 0 | 0 |
| Opitutaceae | 0 | 1 | 0 | 0 | 0 |
| Oxalobacteraceae | 0 | 0 | 0 | 0 | 1 |
| Parachlamydia | 1 | 0 | 0 | 0 | 0 |
| Parasutterella | 0 | 0 | 1 | 0 | 0 |
| Pelagibacterium | 0 | 1 | 0 | 0 | 0 |
| Pelodictyon | 0 | 0 | 0 | 0 | 1 |
| Persephonella | 1 | 0 | 0 | 0 | 0 |
| Pseudogulbenkiania | 0 | 1 | 0 | 0 | 0 |
| Ramlibacter | 1 | 0 | 0 | 0 | 0 |
| Roseiflexus | 0 | 0 | 0 | 0 | 1 |
| Ruegeria | 0 | 1 | 0 | 0 | 0 |
| Serratia | 0 | 1 | 0 | 0 | 0 |
| Silicibacter | 0 | 0 | 0 | 0 | 1 |
| Sinorhizobium | 0 | 0 | 0 | 0 | 1 |
| Sphaerobacter | 0 | 0 | 1 | 0 | 0 |
| Sphingobium | 1 | 0 | 0 | 0 | 0 |
| Sphingomonas | 0 | 0 | 0 | 0 | 1 |
| Spirochaeta | 1 | 0 | 0 | 0 | 0 |
| Syntrophus | 1 | 0 | 0 | 0 | 0 |
| Teredinibacter | 1 | 0 | 0 | 0 | 0 |
| Terriglobus | 0 | 0 | 0 | 1 | 0 |
| Thermoanaerobacterium | 0 | 1 | 0 | 0 | 0 |
| Thermobaculum | 1 | 0 | 0 | 0 | 0 |
| Thioalkalimicrobium | 1 | 0 | 0 | 0 | 0 |
| Thiocapsa | 0 | 1 | 0 | 0 | 0 |
| uncultured bacterium 2304 | 0 | 0 | 1 | 0 | 0 |
| uncultured bacterium 6254 | 0 | 0 | 0 | 0 | 1 |
| uncultured Chloroflexi bacterium | 0 | 1 | 0 | 0 | 0 |
| Verminephrobacter | 0 | 0 | 1 | 0 | 0 |
| Vibrio | 0 | 1 | 0 | 0 | 0 |
| Xanthobacter | 0 | 0 | 0 | 0 | 1 |
| Zobellia | 0 | 1 | 0 | 0 | 0 |
| [Cellvibrio] | 0 | 0 | 0 | 0 | 1 |

Supplemental Table 6. Count of genes annotated as NO reductase classified at the genus level.

| Genus | Un-amended | +O_2_-NO_3_^-^ | +O_2_+NO_3_^-^ | -O_2_-NO_3_^-^ | -O_2_+NO_3_^-^ |
| --- | --- | --- | --- | --- | --- |
| Methylotenera | 21 | 101 | 53 | 1 | 8 |
| Candidatus Nitrospira defluvii | 19 | 3 | 1 | 1 | 6 |
| Methylomonas | 8 | 9 | 10 | 0 | 2 |
| Methylobacter | 7 | 5 | 12 | 0 | 4 |
| Thiobacillus | 4 | 12 | 1 | 0 | 8 |
| Methylomicrobium | 0 | 9 | 8 | 0 | 4 |
| Azoarcus | 5 | 10 | 3 | 0 | 2 |
| Sideroxydans | 10 | 7 | 2 | 0 | 1 |
| Anaerophaga | 9 | 0 | 0 | 0 | 8 |
| uncultured bacterium | 9 | 2 | 2 | 0 | 3 |
| Dechlorosoma | 4 | 4 | 6 | 0 | 1 |
| Geobacter | 6 | 3 | 0 | 1 | 3 |
| Acidovorax | 1 | 0 | 7 | 0 | 3 |
| Bdellovibrio | 8 | 0 | 1 | 0 | 1 |
| Candidatus Solibacter usitatus Ellin6076 | 6 | 2 | 0 | 1 | 1 |
| Gallionella | 4 | 4 | 0 | 0 | 2 |
| Aromatoleum | 2 | 5 | 2 | 0 | 0 |
| endosymbiont | 4 | 4 | 1 | 0 | 0 |
| Leptonema | 3 | 3 | 1 | 0 | 2 |
| Anaeromyxobacter | 5 | 2 | 0 | 0 | 1 |
| Polaromonas | 2 | 0 | 3 | 2 | 1 |
| Thauera | 3 | 4 | 1 | 0 | 0 |
| Leptothrix | 3 | 2 | 1 | 0 | 1 |
| Maribacter | 1 | 2 | 0 | 0 | 4 |
| Mariprofundus | 3 | 3 | 0 | 0 | 1 |
| Sorangium | 2 | 1 | 1 | 0 | 2 |
| Thioalkalivibrio | 1 | 2 | 0 | 1 | 2 |
| uncultured Desulfobacterium sp. | 2 | 1 | 0 | 0 | 3 |
| Candidatus Chloracidobacterium thermophilum B | 2 | 2 | 1 | 0 | 0 |
| Halothiobacillus | 3 | 1 | 1 | 0 | 0 |
| Lutiella | 0 | 2 | 2 | 0 | 1 |
| Muricauda | 2 | 1 | 0 | 0 | 2 |
| Rhodoferax | 0 | 0 | 1 | 1 | 3 |
| Thiothrix | 2 | 2 | 0 | 0 | 1 |
| Candidatus Nitrosoarchaeum limnia SFB1 | 2 | 2 | 0 | 0 | 0 |
| Cupriavidus | 1 | 0 | 0 | 1 | 2 |
| Dechloromonas | 0 | 0 | 0 | 0 | 4 |
| Desulfomonile | 2 | 0 | 0 | 1 | 1 |
| Leptospira | 2 | 1 | 0 | 0 | 1 |
| Nitrosospira | 0 | 1 | 0 | 0 | 3 |
| Acidithiobacillus | 1 | 1 | 1 | 0 | 0 |
| Herminiimonas | 0 | 0 | 3 | 0 | 0 |
| NC10 | 1 | 1 | 1 | 0 | 0 |
| Ralstonia | 0 | 1 | 1 | 0 | 1 |
| Thiomicrospira | 1 | 2 | 0 | 0 | 0 |
| Acaryochloris | 0 | 0 | 0 | 0 | 2 |
| Candidatus Nitrosoarchaeum koreensis MY1 | 0 | 0 | 0 | 1 | 1 |
| Clostridium | 0 | 0 | 0 | 0 | 2 |
| Desulfatibacillum | 2 | 0 | 0 | 0 | 0 |
| Desulfococcus | 1 | 0 | 0 | 1 | 0 |
| Endoriftia | 0 | 0 | 2 | 0 | 0 |
| Haliangium | 2 | 0 | 0 | 0 | 0 |
| Hydrogenovibrio | 1 | 1 | 0 | 0 | 0 |
| Magnetospirillum | 0 | 2 | 0 | 0 | 0 |
| marine | 0 | 1 | 0 | 0 | 1 |
| Pseudogulbenkiania | 1 | 0 | 1 | 0 | 0 |
| Pseudomonas | 1 | 0 | 0 | 0 | 1 |
| Rhodobacter | 0 | 1 | 0 | 0 | 1 |
| Rhodospirillum | 0 | 0 | 0 | 0 | 2 |
| Rubrivivax | 0 | 0 | 2 | 0 | 0 |
| Sulfuricurvum | 2 | 0 | 0 | 0 | 0 |
| Thiomonas | 0 | 2 | 0 | 0 | 0 |
| uncultured archaeon | 2 | 0 | 0 | 0 | 0 |
| Xanthobacter | 0 | 0 | 2 | 0 | 0 |
| Aeromonas | 1 | 0 | 0 | 0 | 0 |
| Azospirillum | 0 | 1 | 0 | 0 | 0 |
| Bacillus | 0 | 0 | 0 | 1 | 0 |
| Bizionia | 0 | 0 | 0 | 0 | 1 |
| Brachyspira | 0 | 0 | 0 | 0 | 1 |
| Burkholderia | 1 | 0 | 0 | 0 | 0 |
| Candidatus Accumulibacter phosphatis clade IIA str. UW-1 | 0 | 0 | 0 | 0 | 1 |
| Candidatus Kuenenia stuttgartiensis | 1 | 0 | 0 | 0 | 0 |
| Chromobacterium | 1 | 0 | 0 | 0 | 0 |
| delta | 1 | 0 | 0 | 0 | 0 |
| Desulfarculus | 0 | 0 | 0 | 1 | 0 |
| Desulfobacula | 1 | 0 | 0 | 0 | 0 |
| Desulfosporosinus | 0 | 0 | 0 | 1 | 0 |
| Desulfurivibrio | 0 | 0 | 0 | 0 | 1 |
| Ectothiorhodospira | 1 | 0 | 0 | 0 | 0 |
| Frankia | 1 | 0 | 0 | 0 | 0 |
| gamma | 1 | 0 | 0 | 0 | 0 |
| Haliscomenobacter | 1 | 0 | 0 | 0 | 0 |
| Halorhodospira | 1 | 0 | 0 | 0 | 0 |
| Holophaga | 0 | 1 | 0 | 0 | 0 |
| Hylemonella | 1 | 0 | 0 | 0 | 0 |
| Ignavibacterium | 0 | 0 | 0 | 0 | 1 |
| Isosphaera | 0 | 1 | 0 | 0 | 0 |
| Marivirga | 0 | 0 | 0 | 0 | 1 |
| Methanohalobium | 0 | 0 | 0 | 0 | 1 |
| Methylocella | 0 | 1 | 0 | 0 | 0 |
| Methylococcus | 0 | 1 | 0 | 0 | 0 |
| Methylophaga | 0 | 1 | 0 | 0 | 0 |
| Methyloversatilis | 1 | 0 | 0 | 0 | 0 |
| Mycobacterium | 0 | 0 | 0 | 1 | 0 |
| Myroides | 0 | 0 | 0 | 0 | 1 |
| Nitrosococcus | 0 | 0 | 0 | 1 | 0 |
| Nitrosopumilus | 0 | 1 | 0 | 0 | 0 |
| Novosphingobium | 0 | 0 | 0 | 0 | 1 |
| Parachlamydia | 0 | 0 | 0 | 0 | 1 |
| Pedosphaera | 0 | 0 | 0 | 1 | 0 |
| Pirellula | 1 | 0 | 0 | 0 | 0 |
| Polymorphum | 0 | 0 | 1 | 0 | 0 |
| Rhodanobacter | 0 | 0 | 0 | 0 | 1 |
| Roseibium | 1 | 0 | 0 | 0 | 0 |
| Shewanella | 0 | 1 | 0 | 0 | 0 |
| Singulisphaera | 0 | 0 | 0 | 0 | 1 |
| Sinorhizobium | 0 | 1 | 0 | 0 | 0 |
| Solemya | 0 | 0 | 0 | 0 | 1 |
| Sphingomonas | 0 | 0 | 0 | 0 | 1 |
| Spirochaeta | 0 | 1 | 0 | 0 | 0 |
| Syntrophobacter | 0 | 0 | 0 | 0 | 1 |
| Thiocapsa | 0 | 1 | 0 | 0 | 0 |
| Thiocystis | 0 | 0 | 0 | 0 | 1 |
| Thiorhodococcus | 1 | 0 | 0 | 0 | 0 |
| Tolumonas | 0 | 0 | 0 | 0 | 1 |
| uncultured organism | 0 | 0 | 0 | 1 | 0 |
| Verrucomicrobiae | 1 | 0 | 0 | 0 | 0 |
| Vibrio | 0 | 0 | 0 | 0 | 1 |

Supplemental Table 7. Count of genes annotated as N_2_O reductase classified at the genus level.

| Genus | Un-amended | +O_2_-NO_3_^-^ | +O_2_+NO_3_^-^ | -O_2_-NO_3_^-^ | -O_2_+NO_3_^-^ |
| --- | --- | --- | --- | --- | --- |
| uncultured bacterium | 26 | 20 | 7 | 7 | 23 |
| Anaeromyxobacter | 8 | 0 | 0 | 2 | 10 |
| Niastella | 5 | 4 | 0 | 0 | 5 |
| uncultured candidate division OP1 bacterium | 9 | 0 | 0 | 0 | 3 |
| Dyadobacter | 2 | 4 | 0 | 1 | 3 |
| Gemmatimonas | 6 | 0 | 0 | 0 | 3 |
| Pseudogulbenkiania | 1 | 7 | 1 | 0 | 0 |
| Rhodothermus | 2 | 1 | 0 | 0 | 6 |
| Runella | 4 | 3 | 0 | 0 | 2 |
| Dechlorosoma | 3 | 1 | 0 | 0 | 3 |
| Flavobacterium | 0 | 3 | 0 | 1 | 3 |
| Caldilinea | 6 | 0 | 0 | 0 | 0 |
| Solitalea | 3 | 0 | 0 | 0 | 3 |
| Candidatus Accumulibacter phosphatis clade IIA str. UW-1 | 3 | 0 | 0 | 0 | 2 |
| Rhodoferax | 1 | 3 | 0 | 0 | 1 |
| Thiobacillus | 0 | 4 | 1 | 0 | 0 |
| Capnocytophaga | 1 | 1 | 0 | 1 | 1 |
| Dechloromonas | 0 | 2 | 1 | 0 | 1 |
| Niabella | 1 | 1 | 0 | 0 | 2 |
| Persephonella | 4 | 0 | 0 | 0 | 0 |
| Thermomicrobium | 1 | 1 | 0 | 0 | 2 |
| Acidovorax | 0 | 2 | 0 | 0 | 1 |
| Azoarcus | 0 | 3 | 0 | 0 | 0 |
| Ferroglobus | 1 | 0 | 0 | 0 | 2 |
| Ignavibacterium | 2 | 1 | 0 | 0 | 0 |
| Leptothrix | 1 | 0 | 1 | 0 | 1 |
| Methylocystis | 0 | 3 | 0 | 0 | 0 |
| Alicycliphilus | 0 | 0 | 0 | 0 | 2 |
| Haliscomenobacter | 1 | 1 | 0 | 0 | 0 |
| Hydrogenobacter | 2 | 0 | 0 | 0 | 0 |
| Lutiella | 0 | 2 | 0 | 0 | 0 |
| Prevotella | 0 | 1 | 0 | 0 | 1 |
| Ralstonia | 0 | 1 | 0 | 1 | 0 |
| Riemerella | 1 | 0 | 0 | 0 | 1 |
| Sphaerobacter | 0 | 1 | 0 | 0 | 1 |
| Aequorivita | 1 | 0 | 0 | 0 | 0 |
| Aromatoleum | 0 | 1 | 0 | 0 | 0 |
| Belliella | 1 | 0 | 0 | 0 | 0 |
| Burkholderia | 0 | 0 | 1 | 0 | 0 |
| Campylobacter | 1 | 0 | 0 | 0 | 0 |
| Cupriavidus | 0 | 1 | 0 | 0 | 0 |
| endosymbiont | 0 | 0 | 0 | 0 | 1 |
| Hydrogenivirga | 1 | 0 | 0 | 0 | 0 |
| Ilyobacter | 0 | 0 | 0 | 1 | 0 |
| Leptospira | 0 | 1 | 0 | 0 | 0 |
| Myroides | 1 | 0 | 0 | 0 | 0 |
| Opitutus | 1 | 0 | 0 | 0 | 0 |
| Owenweeksia | 1 | 0 | 0 | 0 | 0 |
| Pedobacter | 0 | 0 | 0 | 1 | 0 |
| Rubrivivax | 1 | 0 | 0 | 0 | 0 |
| uncultured bacterium 878 | 0 | 1 | 0 | 0 | 0 |

Supplemental Table 8. Count of genes annotated as nitrogenase classified at the genus level.

| Genus | Un-amended | +O_2_-NO_3_^-^ | +O_2_+NO_3_^-^ | -O_2_-NO_3_^-^ | -O_2_+NO_3_^-^ |
| --- | --- | --- | --- | --- | --- |
| Methylobacter | 104 | 194 | 79 | 3 | 23 |
| Methylomonas | 10 | 22 | 20 | 0 | 2 |
| Methylocystis | 1 | 35 | 0 | 0 | 0 |
| Methylococcus | 2 | 20 | 1 | 0 | 1 |
| Methylosinus | 1 | 21 | 0 | 0 | 0 |
| Azoarcus | 1 | 10 | 2 | 0 | 1 |
| Methylocella | 0 | 10 | 1 | 0 | 0 |
| Polaromonas | 1 | 0 | 7 | 0 | 2 |
| Bradyrhizobium | 0 | 7 | 1 | 0 | 0 |
| Acidithiobacillus | 1 | 3 | 1 | 0 | 2 |
| Geobacter | 5 | 0 | 0 | 1 | 0 |
| Rhodopseudomonas | 2 | 0 | 2 | 0 | 2 |
| Sideroxydans | 4 | 0 | 0 | 0 | 1 |
| Thermodesulfovibrio | 4 | 0 | 0 | 0 | 1 |
| Beggiatoa | 2 | 1 | 1 | 0 | 0 |
| Beijerinckia | 0 | 3 | 1 | 0 | 0 |
| Novosphingobium | 0 | 4 | 0 | 0 | 0 |
| Opitutaceae | 4 | 0 | 0 | 0 | 0 |
| Burkholderiales | 0 | 2 | 1 | 0 | 0 |
| Leptothrix | 2 | 0 | 0 | 0 | 1 |
| Methanoregula | 2 | 0 | 0 | 1 | 0 |
| Methanotorris | 2 | 1 | 0 | 0 | 0 |
| Pelobacter | 2 | 0 | 1 | 0 | 0 |
| Tolumonas | 1 | 2 | 0 | 0 | 0 |
| uncultured bacterium | 1 | 2 | 0 | 0 | 0 |
| Xanthobacter | 0 | 3 | 0 | 0 | 0 |
| Anaeromyxobacter | 1 | 0 | 0 | 0 | 1 |
| Azospirillum | 1 | 1 | 0 | 0 | 0 |
| Burkholderia | 0 | 0 | 2 | 0 | 0 |
| Dechloromonas | 0 | 1 | 0 | 0 | 1 |
| Dehalococcoides | 1 | 0 | 0 | 0 | 1 |
| Rhodobacter | 1 | 0 | 0 | 0 | 1 |
| Syntrophobacter | 1 | 1 | 0 | 0 | 0 |
| Thiothrix | 2 | 0 | 0 | 0 | 0 |
| Azorhizobium | 0 | 1 | 0 | 0 | 0 |
| Azotobacter | 1 | 0 | 0 | 0 | 0 |
| Candidatus Accumulibacter phosphatis clade IIA str. UW-1 | 0 | 0 | 0 | 0 | 1 |
| Chlorobaculum | 0 | 0 | 0 | 0 | 1 |
| Chlorobium | 0 | 0 | 0 | 1 | 0 |
| Dechlorosoma | 0 | 0 | 1 | 0 | 0 |
| Desulfarculus | 1 | 0 | 0 | 0 | 0 |
| Desulfobacterium | 1 | 0 | 0 | 0 | 0 |
| Desulfobulbus | 1 | 0 | 0 | 0 | 0 |
| Desulfovibrio | 0 | 1 | 0 | 0 | 0 |
| Herbaspirillum | 0 | 0 | 1 | 0 | 0 |
| Hyphomicrobium | 0 | 1 | 0 | 0 | 0 |
| Leptospirillum | 0 | 1 | 0 | 0 | 0 |
| Methanohalophilus | 0 | 0 | 0 | 0 | 1 |
| Methanothermobacter | 0 | 0 | 0 | 0 | 1 |
| Methylobacterium | 1 | 0 | 0 | 0 | 0 |
| Peptoniphilus | 1 | 0 | 0 | 0 | 0 |
| Phascolarctobacterium | 0 | 0 | 0 | 0 | 1 |
| Rhodomicrobium | 0 | 1 | 0 | 0 | 0 |
| Rubrivivax | 0 | 1 | 0 | 0 | 0 |
| Selenomonas | 1 | 0 | 0 | 0 | 0 |
| Sulfuricurvum | 1 | 0 | 0 | 0 | 0 |
| Teredinibacter | 1 | 0 | 0 | 0 | 0 |
| Thiocystis | 0 | 1 | 0 | 0 | 0 |
| Thiorhodococcus | 1 | 0 | 0 | 0 | 0 |
| Thiorhodospira | 1 | 0 | 0 | 0 | 0 |
| Thiorhodovibrio | 0 | 0 | 1 | 0 | 0 |
| Treponema | 0 | 1 | 0 | 0 | 0 |
| uncultured microorganism | 1 | 0 | 0 | 0 | 0 |
| uncultured nitrogen-fixing bacterium | 1 | 0 | 0 | 0 | 0 |
| uncultured nitrogen-fixing bacterium B34 | 0 | 1 | 0 | 0 | 0 |
| uncultured soil bacterium | 0 | 1 | 0 | 0 | 0 |
