# Supplementary material for: A metagenomic insight into freshwater methane-utilizing communities and evidence for cooperation between the Methylococcaceae and the Methylophilaceae
Source: PeerJ. 2013 Feb 19;1:e23. doi: 10.7717/peerj.23 (PMC3628875; doi:10.7717/peerj.23)
Supplement: Supplemental Table 1 [file peerj-01-23-s004.pdf]

Supplemental Table 1. Distribution of 16S rRNA genes among major phyla, classified to the family level

Phylum	Class	Order	Family	Un-amended	$+O_2-NO_3^-$	$+O_2+NO_3^-$	$-O_2-NO_3^-$	$-O_2+NO_3^-$
Proteobacteria	Gammaproteobacteria	Methylococcales	Methylococcaceae	15	26	4	2	6
Bacteroidetes	unclassified Bacteroidetes			23	1	2	2	14
Proteobacteria	Gammaproteobacteria	Acidithiobacillales	Acidithiobacillaceae	0	1	0	0	0
Proteobacteria	Gammaproteobacteria	Thiotrichales	Piscirickettsiaceae	0	1	0	0	0
Actinobacteria	Actinobacteria	Actinobacteridae	unclassified Actinobacteridae	0	0	0	1	0
Cyanobacteria	Cyanobacteria	Family II	GpIIa	0	0	0	2	1
Acidobacteria	Acidobacteria Gp10	Gp10		0	0	0	0	1
Actinobacteria	Actinobacteria	Acidimicrobidae	Acidimicrobiales	0	0	0	0	1
Bacteroidetes	Flavobacteria	Flavobacteriales	Cryomorphaceae	0	0	0	0	2
Chloroflexi	Caldilineae	Caldilineales	Caldilineaceae	0	0	0	0	2
Firmicutes	Clostridia	unclassified Clostridia		0	0	0	0	1
Proteobacteria	Deltaproteobacteria	Bdellovibrionales	Bacteriovoracaceae	0	0	0	0	1
Proteobacteria	Gammaproteobacteria	Pseudomonadales	Pseudomonadaceae	0	0	0	0	2
TM7	TM7 genera incertae sedis			0	0	0	0	2
Euryarchaeota	Methanomicrobia	Methanomicrobiales	Methanomicrobiaceae	1	0	0	0	0
Euryarchaeota	Thermoplasmata	Thermoplasmatales	Thermoplasmatales incertae sedis	2	0	0	0	0
unclassified Archaea				8	0	0	1	4
Acidobacteria	Acidobacteria Gp17	Gp17		3	2	0	1	0
Acidobacteria	Acidobacteria Gp3	Gp3		5	0	0	0	0

Acidobacteria	Acidobacteria Gp6	Gp6		8	0	0	3	4
Acidobacteria	Holophagae	Holophagales	Holophagaceae	4	0	0	1	4
Actinobacteria	Actinobacteria	Actinobacteridae	Actinomycetales	9	2	0	0	3
Actinobacteria	Actinobacteria	Rubrobacteridae	Solirubrobacterales	1	1	0	1	1
Bacteroidetes	Bacteroidetes incertae sedis	Prolixibacter		2	1	0	0	2
Bacteroidetes	Flavobacteria	Flavobacteriales	unclassified Flavobacteriales	1	0	0	0	0
Bacteroidetes	Sphingobacteria	Sphingobacteriales	Sphingobacteriaceae	1	0	0	0	0
Chlamydiae	Chlamydiae	Chlamydiales	unclassified Chlamydiales	1	0	0	0	0
Chloroflexi	Dehalococcoidetes	Dehalogenimonas		3	0	0	1	0
Cyanobacteria	Cyanobacteria	Chloroplast	Bacillariophyta	5	2	0	3	1
Cyanobacteria	Cyanobacteria	Chloroplast	unclassified Chloroplast	1	0	0	0	0
Firmicutes	Clostridia	Clostridiales	Incertae Sedis XI	1	0	0	0	0
Lentisphaerae	Lentisphaeria	unclassified Lentisphaeria		1	0	0	0	0
OD1	OD1 genera incertae sedis			4	1	0	0	1
Planctomycetes	Planctomycetacia	Planctomycetales	Planctomycetaceae	11	7	0	1	7
Proteobacteria	Alphaproteobacteria	Rhizobiales	unclassified Rhizobiales	2	2	0	0	1
Proteobacteria	Alphaproteobacteria	Rhodospirillales	Rhodospirillaceae	2	0	0	0	0
Proteobacteria	Betaproteobacteria	Burkholderiales	Burkholderiales incertae sedis	6	3	1	0	2
Proteobacteria	Betaproteobacteria	Burkholderiales	Oxalobacteraceae	5	0	1	0	8
Proteobacteria	Betaproteobacteria	Methylophilales	Methylophilaceae	5	21	1	0	5
Proteobacteria	Betaproteobacteria	Rhodocyclales	Rhodocyclaceae	4	0	0	0	7
Proteobacteria	Deltaproteobacteria	Desulfobacterales	Desulfobacteraceae	1	1	0	1	3
Proteobacteria	Deltaproteobacteria	Desulfuromonadales	Geobacteraceae	2	1	0	0	0
Proteobacteria	Deltaproteobacteria	Myxococcales	Nannocystineae	3	0	0	0	0
Proteobacteria	Deltaproteobacteria	Myxococcales	unclassified Myxococcales	2	3	0	2	0
Proteobacteria	Deltaproteobacteria	unclassified Deltaproteobacteria		9	4	0	0	7

Proteobacteria	Epsilonproteobacteria	Campylobacterales	Helicobacteraceae	3	0	0	1	2
Proteobacteria	Gammaproteobacteria	Xanthomonadales	Sinobacteraceae	4	2	0	1	2
Proteobacteria	unclassified Proteobacteria			9	6	0	0	6
Spirochaetes	Spirochaetes	Spirochaetales	Spirochaetaceae	2	0	0	0	0
Verrucomicrobia	Opitutae	Opitutales	Opitutaceae	2	0	0	0	2
Verrucomicrobia	Subdivision3	Subdivision3 genera incertae sedis		13	3	0	0	3
Verrucomicrobia	unclassified Verrucomicrobia			8	0	0	1	6
WS3	WS3 genera incertae sedis			2	0	0	0	0
Actinobacteria	Actinobacteria	Rubrobacteridae	unclassified Rubrobacteridae	0	1	0	0	0
Firmicutes	Clostridia	Clostridiales	Ruminococcaceae	0	1	0	1	0
Proteobacteria	Alphaproteobacteria	Rhizobiales	Hyphomicrobiaceae	0	1	0	0	1
Proteobacteria	Alphaproteobacteria	Rickettsiales	Anaplasmataceae	0	1	0	0	0
Proteobacteria	Gammaproteobacteria	Pseudomonadales	Moraxellaceae	0	2	0	0	0
Euryarchaeota	Thermoplasmata	Thermoplasmatales	unclassified Thermoplasmatales	0	0	0	1	0
Chlamydiae	Chlamydiae	Chlamydiales	Waddliaceae	0	0	0	1	0
OP11	OP11 genera incertae sedis			0	0	0	1	0
Acidobacteria	Acidobacteria Gp11	Gp11		0	0	0	0	1
Bacteroidetes	Bacteroidetes incertae sedis	unclassified Bacteroidetes incertae sedis		0	0	0	0	1
Bacteroidetes	Sphingobacteria	Sphingobacteriales	Saprospiraceae	0	0	0	0	2
Cyanobacteria	Cyanobacteria	unclassified		0	0	0	0	1

		Cyanobacteria						
Proteobacteria	Alphaproteobacteria	Sphingomonadales	unclassified Sphingomonadales	0	0	0	0	1
Proteobacteria	Gammaproteobacteria	Legionellales	Legionellaceae	0	0	0	0	1
Proteobacteria	Gammaproteobacteria	Thiotrichales	Thiotrichaceae	0	0	0	0	1
Euryarchaeota	Methanomicrobia	Methanomicrobiales	unclassified Methanomicrobiales	1	0	0	0	0
Euryarchaeota	unclassified Euryarchaeota			13	3	0	1	2
Acidobacteria	Acidobacteria Gp16	Gp16		3	1	0	0	2
Acidobacteria	Acidobacteria Gp22	Gp22		3	0	0	0	0
Acidobacteria	Acidobacteria Gp4	Gp4		4	0	0	0	1
Acidobacteria	Acidobacteria Gp7	Gp7		1	0	0	0	4
Actinobacteria	Actinobacteria	Acidimicrobidae	unclassified Acidimicrobidae	1	0	0	0	1
Actinobacteria	Actinobacteria	Coriobacteridae	Coriobacteriales	3	1	0	0	1
Actinobacteria	Actinobacteria	unclassified Actinobacteria		4	0	0	0	4
Bacteroidetes	Flavobacteria	Flavobacteriales	Flavobacteriaceae	1	2	0	0	2
Bacteroidetes	Sphingobacteria	Sphingobacteriales	Chitinophagaceae	7	2	0	0	3
Bacteroidetes	Sphingobacteria	Sphingobacteriales	unclassified Sphingobacteriales	4	1	0	0	5
Chlamydiae	Chlamydiae	Chlamydiales	Parachlamydiaceae	1	0	0	0	2
Chloroflexi	Anaerolineae	Anaerolineales	Anaerolineaceae	17	1	0	2	5
Chloroflexi	unclassified Chloroflexi			2	2	0	0	3
Cyanobacteria	Cyanobacteria	Chloroplast	Chlorophyta	1	0	0	0	0
Firmicutes	Bacilli	Bacillales	Bacillaceae	1	0	0	0	0
Gemmatimonadetes	Gemmatimonadetes	Gemmatimonadales	Gemmatimonadaceae	8	2	0	1	1
Nitrospira	Nitrospira	Nitrospirales	Nitrospiraceae	3	0	1	1	2

OP10	OP10 genera incertae sedis			1	0	0	0	0
Proteobacteria	Alphaproteobacteria	Caulobacterales	Caulobacteraceae	1	1	0	0	0
Proteobacteria	Alphaproteobacteria	Rhizobiales	Xanthobacteraceae	1	0	0	0	0
Proteobacteria	Alphaproteobacteria	unclassified Alphaproteobacteria		6	0	0	1	0
Proteobacteria	Betaproteobacteria	Burkholderiales	Comamonadaceae	3	0	4	1	5
Proteobacteria	Betaproteobacteria	Burkholderiales	unclassified Burkholderiales	2	3	0	0	1
Proteobacteria	Betaproteobacteria	Nitrosomonadales	Nitrosomonadaceae	4	0	0	0	0
Proteobacteria	Betaproteobacteria	unclassified Betaproteobacteria		15	16	0	1	8
Proteobacteria	Deltaproteobacteria	Desulfobacterales	Desulfobulbaceae	4	0	0	1	0
Proteobacteria	Deltaproteobacteria	Myxococcales	Cystobacterineae	3	3	0	0	1
Proteobacteria	Deltaproteobacteria	Myxococcales	Sorangiineae	4	5	0	0	1
Proteobacteria	Deltaproteobacteria	Syntrophobacterales	Syntrophaceae	1	0	0	1	1
Proteobacteria	Epsilonproteobacteria	Campylobacterales	Campylobacteraceae	1	0	0	0	0
Proteobacteria	Gammaproteobacteria	Enterobacteriales	Enterobacteriaceae	1	0	0	0	0
Proteobacteria	Gammaproteobacteria	unclassified Gammaproteobacteria		11	8	1	0	5
Proteobacteria	Gammaproteobacteria	Xanthomonadales	Xanthomonadaceae	1	0	0	0	4
Spirochaetes	Spirochaetes	Spirochaetales	Leptospiraceae	12	0	2	2	3
Verrucomicrobia	Spartobacteria	Spartobacteria genera incertae sedis		1	0	0	0	0
Verrucomicrobia	Subdivision5	Subdivision5 genera incertae sedis		4	0	0	0	2
Verrucomicrobia	Verrucomicrobiae	Verrucomicrobiales	Verrucomicrobiaceae	2	1	0	1	1
Euryarchaeota	Methanomicrobia	Methanosarcinales	Methanosaetaceae	0	1	0	0	3
Firmicutes	Clostridia	Clostridiales	Incertae Sedis XIII	0	2	0	0	3

Firmicutes	Clostridia	Clostridiales	unclassified Clostridiales	0	1	0	0	1
Proteobacteria	Alphaproteobacteria	Rhizobiales	Methylocystaceae	0	4	2	0	0
Proteobacteria	Deltaproteobacteria	Bdellovibrionales	Bdellovibrionaceae	0	1	0	0	0
